# Assessing maladaptive sugar-rich food consumption: development and preliminary validation of the sugar misuse questionnaire

**DOI:** 10.3389/fnut.2026.1855019

**Published:** 2026-08-07

**Authors:** Pascal Perney, Raphaël Trouillet, Sophie Schuldiner, Hélène Donnadieu, Philippe Lehert, Mickael Naassila

**Affiliations:** 1 Addictions Department, CHU Caremeau, University of Montpellier, Montpellier, France; 2 INSERM U1018, Paris, France; 3 University Paul Valéry Montpellier 3, University Montpellier, EPSYLON EA 4556, Montpellier, France; 4 VBIC, INSERM U1047, Université de Montpellier, Service des Maladies Métaboliques et Endocriniennes, CHU Nîmes, Clinique du Pied Gard Occitanie, Le Grau du Roi, France; 5 Addictions Department, Hôpital La Colombiere, University Hospital of Montpellier, Inserm U1058, Montpellier, France; 6 Montpellier Faculty of Medicine, University of Montpellier, Montpellier, France; 7 University of Melbourne, Parkville, VIC, Australia; 8 Université Catholique de Louvain, Ottignies-Louvain-la-Neuve, Belgium; 9 Groupe de Recherche sur l'Alcool & les Pharmacodépendances GRAP, INSERM UMR 1247, Université de Picardie Jules Verne, Amiens, France; 10 Institut Universitaire de France, Paris, France

**Keywords:** addiction, eating behavior, psychometrics, questionnaire, screening, sugar misuse, sugar-rich foods

## Abstract

**Objective:**

Excessive consumption of sugar-rich foods is associated with adverse metabolic and health outcomes. Beyond the amount of sugar consumed, growing evidence suggests that some individuals experience maladaptive patterns of sugar-rich food consumption characterized by craving, loss of control, and persistence despite negative consequences. However, existing instruments primarily assess general food addiction or eating disorders and may not adequately capture behavioral patterns specifically related to sugar-rich products.

**Method:**

The sugar misuse questionnaire (SuMQ) was developed using a mixed-methods approach. First, a qualitative study was conducted with individuals reporting severe difficulties related to sugar-rich food consumption to identify salient experiential dimensions of sugar misuse (SuM). Key themes were extracted through thematic analysis and used to generate questionnaire items. Second, the psychometric properties of the SuMQ were evaluated in a general population sample. Item functioning and scale structure were examined using Rasch modeling, followed by dimensionality analyses. ROC analyses were conducted to explore preliminary severity thresholds.

**Results:**

Qualitative analyses identified 25 recurrent concepts related to maladaptive sugar-rich food consumption, which informed the initial version of the questionnaire. Rasch analysis supported a reduced 11-item version with strong internal consistency and a unidimensional structure. Exploratory threshold analyses identified score ranges associated with higher self-reported severity of SuM.

**Discussion:**

The SuMQ provides a behaviorally grounded, preliminary psychometrically validated instrument for assessing sugar-focused maladaptive eating patterns within a dimensional framework. It may support research exploring the boundaries between addictive-like eating and established eating disorder constructs.

## Introduction

Excessive consumption of sugar-rich foods has become a major public health concern worldwide because of its well-established contribution to obesity, type 2 diabetes, cardiovascular diseases, metabolic dysfunction, and several chronic inflammatory conditions (1–3). Beyond these metabolic consequences, growing evidence indicates that some individuals experience recurrent behavioral difficulties characterized by intense craving, impaired control over consumption, persistent intake despite adverse consequences, and repeated unsuccessful attempts to reduce sugar-rich food consumption. These behavioral manifestations have attracted increasing attention because they resemble dimensions commonly described in addictive disorders while remaining embedded within eating behavior.

The conceptual interpretation of these behaviors remains debated. Several overlapping frameworks have been proposed, including food addiction, sugar addiction, and more recently, Ultra-Processed Food Use Disorder (UPFUD) (4–6). Although these models differ in their theoretical assumptions and diagnostic implications, they all attempt to describe maladaptive patterns of consumption that extend beyond simple dietary preference or overeating. At present, however, no consensus exists regarding the boundaries separating these constructs or their clinical significance.

In the present research, we use the term sugar misuse (SuM) to describe a dimensional behavioral construct characterized by maladaptive consumption of sugar-rich foods associated with impaired control, recurrent craving, persistence despite adverse consequences, and compulsive behavioral features, without implying the existence of a distinct addictive disorder. Rather than proposing a new diagnosis, SuM is conceptualized as a measurable behavioral phenotype that may facilitate clinical characterization and research independently of ongoing nosological debates. Operationally, SuM refers to the measurable behavioral manifestations of this phenotype, whereas the sugar misuse questionnaire (SuMQ) is the psychometric instrument specifically developed to operationalize and quantify this construct.

This conceptual distinction has important methodological implications. Existing questionnaires were primarily designed to assess food addiction according to addiction criteria, eating disorders, sweet taste preference, or problematic consumption of ultra-processed foods. To our knowledge, no instrument has been specifically developed to quantify maladaptive sugar-rich food consumption as an independent behavioral construct. This measurement gap provided the rationale for developing the SuMQ.

**Figure 1:**
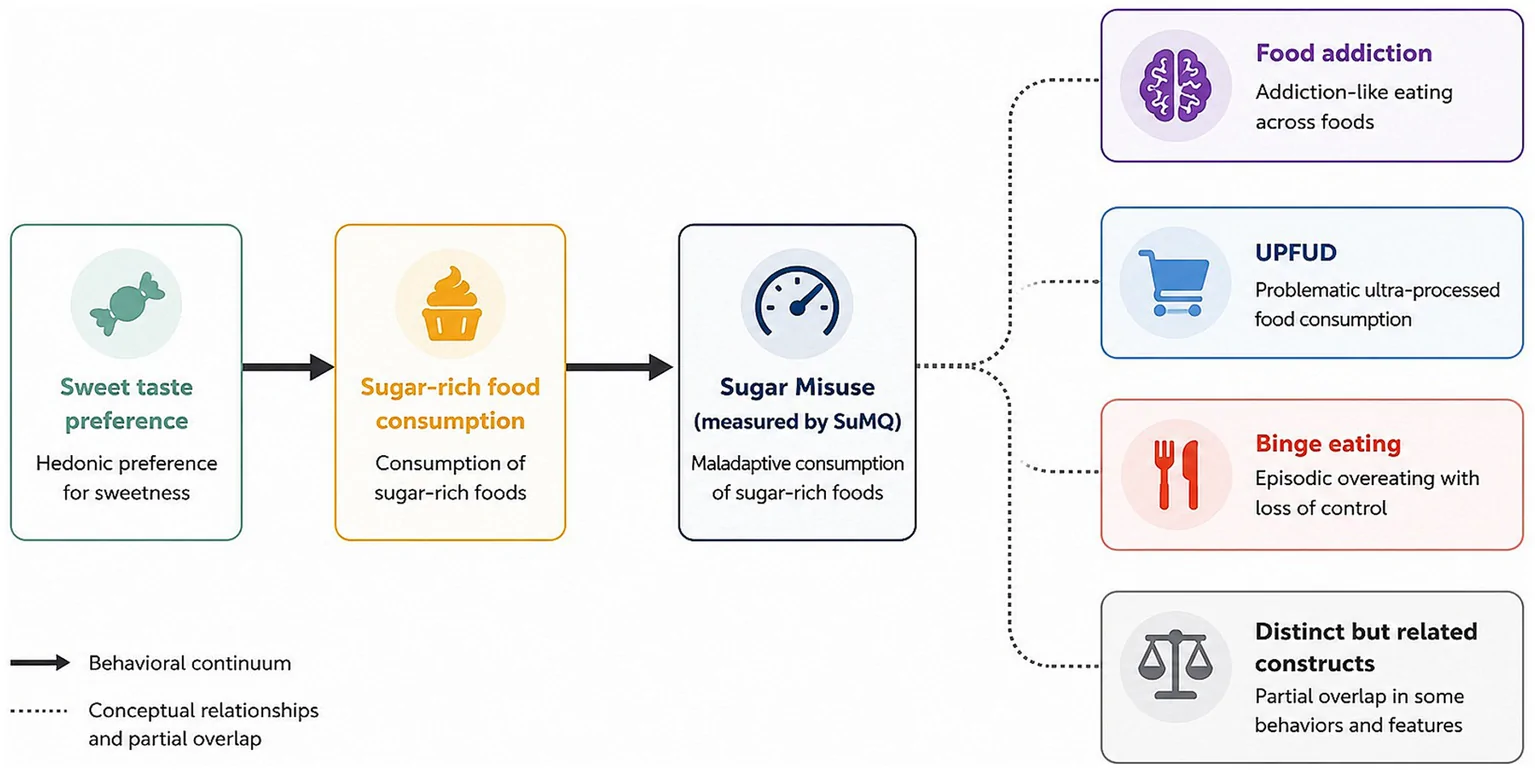
Conceptual positioning of sugar misuse within related constructs of eating behavior. SuM is conceptualized as a dimensional behavioral phenotype that describes maladaptive consumption of sugar-rich foods characterized by impaired control, craving, persistence despite adverse consequences, and other behavioral manifestations. The solid arrows represent a behavioral continuum, extending from sweet taste preference to sugar-rich food consumption and, in a subset of individuals, to SuM. Dashed lines indicate conceptual overlap rather than diagnostic equivalence.

To further clarify the conceptual positioning of SuM relative to neighboring constructs, Table 1 summarizes the main conceptual similarities and distinctions between SuM, food addiction, sugar addiction, binge-eating, sweet taste preference, and Ultra-Processed Food Use Disorder (UPFUD). Figure 1 illustrates the proposed conceptual framework underlying the development of the SuMQ.

**Table 1:** Conceptual positioning of sugar misuse among related eating behavior constructs.

Construct	Primary focus	Conceptual framework	Diagnostic status	Typical assessment
Sweet taste preference	Hedonic preference for sweetness	Sensory/hedonic	Not pathological	Sweet Taste Questionnaire
Binge-eating	Episodic overeating with loss of control	Eating disorder	DSM-5 diagnosis	BES, EDE-Q
Food addiction	Addiction-like eating across foods	Addiction framework	Controversial	YFAS-2.0
Ultra-Processed Food Use Disorder (UPFUD)	Problematic ultra-processed food consumption	Emerging addiction-related framework	Emerging construct	UPFUD instruments
Sugar misuse (SuM)	Maladaptive consumption of sugar-rich foods	Dimensional behavioral phenotype	Behavioral construct	SuMQ

SuM is conceptualized as a dimensional behavioral phenotype that describes the maladaptive sugar-rich food consumption. The table summarizes the conceptual similarities and distinctions between SuM and adjacent constructs, including sweet taste preference, binge-eating, food addiction, and ultra-processed food use disorder (UPFUD). The SuMQ was specifically developed to operationalize SuM rather than diagnose existing psychiatric disorders.

The first rationale for addressing excessive sugar consumption is its well-documented contribution to morbidity and mortality. Diets with a high glycemic index have been associated with increased risk of type 2 diabetes mellitus (T2DM) (7). Historical data from the United States demonstrate parallel trends between rising added sugar intake and the epidemics of obesity and T2DM (3). Sugar-sweetened beverages (SSBs), in particular, are linked to a dose-dependent increase in T2DM risk (8) and are major contributors to overweight and obesity in both adults and children (9).

Analysis of the NHANES III mortality cohort has shown that added sugar and SSBs consumption are associated with arterial hypertension, stroke, coronary heart disease, and dyslipidemia, thereby increasing overall mortality risk (3, 10). Additional cohort studies have established significant associations between SSBs intake and metabolic syndrome, a precursor to cardiometabolic disease (10). Moreover, recent research has identified links between high-sugar diets and chronic inflammatory conditions such as rheumatoid arthritis, multiple sclerosis, psoriasis, and inflammatory bowel disease (10). Finally, both T2DM and high SSB consumption have been associated with increased risk, faster progression, and higher mortality in several cancers (3, 11).

The second motivation for early identification of problematic sugar-rich food consumption lies in the extensive public health efforts to curb this behavior. Several studies have shown that reducing sugar-rich food consumption can yield significant health benefits. For instance, in a tightly controlled inpatient feeding study, a low-carbohydrate diet resulted in a significant reduction in mean HbA1c, from 7.3 to 6.8%, after only 14 days (7). Such dietary interventions often allow for a reduction in diabetes medication under clinical supervision.

There is broad consensus on the need to reduce or eliminate processed red meats, refined grains, and added sugars (especially SSBs) for both prevention and management of T2DM (7). The World Health Organization (WHO) recommends limiting added sugar-rich food consumption to less than 10% of total energy intake, and sugar reduction could also decrease the risk of dementia and “type 3 Diabetes mellitus” (12).

Although the health risks associated with excessive sugar-rich food consumption are well documented, modifying this behavior remains a complex challenge. This highlights the third reason for understanding SuM: the need to better characterize individuals with excessive sugar-rich food consumption. Much of the current literature on high-sugar foods is grounded in theoretical addiction models or relies on animal and neuroimaging studies. However, little attention has been paid to the knowledge of those directly affected, individuals with SuM, despite their potential to offer invaluable insight into their own behaviors. For example, alcohol-dependent patients report an increase in sugar craving, consumption, and weight during alcohol withdrawal (13, 14). From a clinical perspective, these changes in sugar-rich food consumption are not limited to an increase in the quantity of sugar-rich products ingested. Furthermore, it reflects a new form of sugar-rich food consumption, including craving, storage of sweets, and the loss of control over sugar-rich food consumption, as a more elevated hedonic reaction to sweet taste is associated with an impaired control over eating sweets (13, 15).

Although several instruments have been developed to investigate problematic eating behaviors, none was specifically designed to operationalize SuM as defined in the present study. The Yale Food Addiction Scale (YFAS) and its derivatives were developed to assess addiction-like eating according to DSM substance use disorder criteria and therefore primarily operationalize the food addiction construct rather than sugar-specific maladaptive behaviors (16, 17). Similarly, the sugar addiction questionnaire and the FitMIND Foundation Sweets Addiction Scale were explicitly developed within an addiction framework (18, 19). The Sweet Taste Questionnaire (STQ) primarily assesses sweet taste reward sensitivity, attitudes toward sweet foods, mood-related effects of sweet consumption, and perceived control over eating sweets (15). More recently, instruments assessing UPFUD have broadened their scope toward ultra-processed foods rather than sugar-rich foods specifically (20). Consequently, an important measurement gap remains for an instrument specifically designed to quantify SuM as a dimensional behavioral phenotype grounded in patients’ lived experiences rather than in pre-existing diagnostic frameworks.

The application of addiction frameworks to eating behaviors remains debated. Several authors have emphasized that although certain eating patterns may share phenomenological similarities with Substance use disorder (SUD), including craving and loss of control, they do not necessarily constitute a diagnosable addiction (5). In this context, dimensional approaches that focus on addictive-like eating behaviors rather than categorical diagnoses have been proposed as a more appropriate framework for understanding problematic food consumption.

Recent developments have extended the debate surrounding addictive-like eating behaviors, introducing concepts such as food addiction and, more recently, UPFUD. While some authors argue that highly processed foods may trigger behavioral and neurobiological responses resembling substance-use disorders, others have questioned the conceptual distinctiveness and diagnostic validity of these constructs. In particular, concerns have been raised about the overlap between food addiction, craving, loss of control, emotional eating, and other established eating-related psychopathologies (6). Consequently, the nature and boundaries of addictive-like eating behaviors remain actively debated. Recent consensus-oriented research has also proposed ultra-processed food addiction as a clinically relevant construct, while recent debates have emphasized both the potential usefulness and the conceptual challenges of applying addiction frameworks to food-related behaviors (20, 21).

Experimental studies have shown that intermittent sugar exposure can trigger behavioral and neurobiological changes that resemble some features of addictive processes in animal models (22). More broadly, recent reviews have highlighted how highly processed foods may engage reward-related neurobiological pathways implicated in compulsive consumption (23). However, the extent to which these findings can be generalized to human eating behaviors remains controversial (24).

Overall, the available literature supports two major conclusions. First, maladaptive patterns of sugar-rich food consumption represent clinically meaningful behavioral phenomena that deserve specific investigation. Second, despite the availability of several questionnaires assessing food addiction, binge-eating, or related constructs, no instrument has been specifically designed to operationalize SuM as a dimensional behavioral phenotype grounded in patients’ lived experiences. This measurement gap provided the rationale for developing the SuMQ.

The present study aimed to develop the SuMQ, a psychometric instrument designed to operationalize SuM as a dimensional behavioral construct grounded in patients’ lived experiences. Using a sequential mixed-methods approach, we first identified the core phenomenological dimensions of SuM through qualitative interviews with individuals reporting severe sugar-related difficulties. These findings informed the development of questionnaire items, which were then refined and psychometrically evaluated using Rasch modeling, dimensionality analyses, and confirmatory factor analysis. Finally, we explored preliminary research thresholds to support future clinical characterization, epidemiological investigations, and longitudinal intervention studies.

## Methods

### Ethics statement

This study was conducted in accordance with the principles of the Declaration of Helsinki. Ethical approval was obtained from the Direction de la Recherche Clinique et de l’Innovation (DRCI) of Nîmes University Hospital (CHU de Nîmes). The study protocol was reviewed and assigned an approval code by the Institutional Review Board (IRB no. 26.01.08). The Institutional Review Board (IRB no. 26.01.08) is an independent ethics committee responsible for reviewing research involving human participants at CHU de Nîmes. All participants provided informed consent prior to inclusion in the study. Data were collected and processed in compliance with applicable French regulations regarding research involving human participants. Data were collected anonymously and analyzed in accordance with applicable data protection regulations.

Patients from addiction and diabetology units who self-identified as being “addicted to” or having a “specific craving for” sugar-rich products were invited to participate in interviews exploring their potential SuM. This deliberate focus on individuals reporting the most severe forms of sugar-related difficulties was chosen to maximize the identification of salient and clinically meaningful features of SuM, rather than to estimate prevalence or represent the full spectrum of sugar-rich food consumption behaviors. Such an approach is consistent with qualitative methodologies aimed at concept elicitation, in which extreme or information-rich cases are intentionally selected to ensure that the full range of relevant experiential dimensions is captured at early stages of instrument development. Interview guide construction. The interview guide was developed to address key areas presumed to be associated with SuM. For this guide, we used findings from Alarcon et al. (13), who reported an increase in sugar craving in alcohol-dependent patients following alcohol withdrawal (13). Qualitative data were collected by a dietitian and not included in the original quantitative study; they formed the basis of our interview protocol. The face and content validity of our guide were verified by a group of experts in the field of addiction and eating disorders.

Interviews were conducted by clinically trained researchers using a semi-structured guide that began with open-ended questions and progressed to more specific prompts to minimize interviewer bias. The interview was divided into two main parts: the first focused on sugar-rich food consumption in everyday life, including eating habits, regular sugar intake, and the perceived importance of sugar in participants’ lives. The second part explored misuse behaviors, including internal and external triggers, potential withdrawal symptoms, and emotional or functional consequences of sugar-rich food consumption. The final guide included nine themes and 60 possible questions. Of these, 41 questions (68.3%) were derived from prior clinical observations, 12 (20%) were adapted from the YFAS, and 7 (11.7%) from DSM-5 criteria.

Qualitative analysis was conducted by a scientific board composed of physicians and a psychologist. We employed both inductive and deductive content analysis to identify the main dimensions of SuM. Audio recordings were transcribed and analyzed using QDA Miner Lite (Provalis Research, 2016 QDA Miner LITE. Version 2.0.9. https://provalisresearch.com/products/qualitative-data-analysis-software/). Verbatim reports were read in full, then coded line by line. Codes were assigned to data reflecting triggers, consequences, frequency, severity, and outcomes of SuM. When a new concept emerged that was not captured by existing codes, the coding scheme was revised accordingly. This iterative process continued until data saturation was achieved, that is, when no new insights emerged from additional interviews (25). Once saturation was confirmed, two additional interviews were conducted to ensure comprehensiveness. Codes were then grouped into categories based on thematic relationships to form meaningful clusters.

In addition to extracting core themes, we aimed to identify clinically salient features of SuM to formulate questions to be included in our questionnaire. The frequency of each code was calculated as the percentage of participants who mentioned it. Keywords were finally retained according to their frequency. In cases of redundancy between items, we selected the one that best reflected the underlying concept. We also applied the Content Validity Ratio (CVR) method to identify essential items. Traditionally used to assess the relevance of psychometric items, CVR is calculated as: CVR = (*Ne* − *N*/2)/(*N*/2), where *N* is the total number of respondents and *Ne* is the number identifying the item as essential. Based on Lawshe’ s (26) table, a minimum CVR of 0.64 was required, which corresponds to at least 9 out of 10 participants endorsing the item as essential (26).

The final questionnaire was constructed using the most salient codes and themes identified through qualitative analysis. Items were written to capture the principal dimensions of high-sugar food consumption as described by participants (see Table 2 for domains and associated codes). Where applicable, item phrasing was inspired by the YFAS-2.0 and DSM-5 terminology. Each item was rated using a 5-point Likert scale (0 to 4), enabling calculation of a total score ranging from 0 to 100 to quantify SuM. The questionnaire was reviewed by experts in diabetology and addiction, as well as by diabetic patients. They evaluated the items for relevance, clarity, and consistency.

**Table 2:** Conceptual development of sugar misuse (SuM).

Panel A. Conceptual domains identified through qualitative interviews	
Conceptual domain	Definition (based on participant narratives)
Consequences	Emotional, psychological, and functional outcomes attributed to sugar-rich food consumption.
Craving	Persistent urges or desires for sugar-rich products that are difficult to resist.
Loss of control	Difficulty regulating consumption despite intentions or goals.
Tolerance-like experiences	Need for increasing amounts or repeated consumption to obtain similar effects.
Withdrawal-like experiences	Negative emotional or physical states when sugar-rich foods are unavailable.
Predisposing factors	Personal or familial characteristics perceived as increasing vulnerability.
Triggers	Internal or external cues precipitating sugar-rich food consumption.

Panel B. Illustrative participant-derived concepts and representative SuMQ items		
Conceptual domain	Illustrative participant-derived concepts	Representative SuMQ items
Consequences	Shame, negative affects, relaxation, appetite regulation, **pleasure**, **mood enhancement**, **positive affect**, reward, Desire Satisfaction, **guilt**	Q19, Q25
Craving	Uncontrollable urge, obsession, daily desire, **urgency**, **perceived need**, intensive desire, nocturnal consumption, intrusive thoughts	Q5, Q18
Loss of control	Compulsive behavior, **Consumption outside meals**, binge-eating, gorging, finishing what was started, continuous consumption, eating beyond disgust threshold, **consumption despite health consequences**, eating beyond satiety, **excessive consumption duration/amount**	Q8, Q9, Q25
Tolerance-like experiences	Increased sugar preference, **consumption increases craving, and sugar stockpiling**	Q13, Q22
Withdrawal-like experiences	**Withdrawal symptoms**, dysphoria during abstinence, sugar relieves withdrawal, **tension in the absence of sugar**	Q14, Q15
Predisposing factors	Family-degree history of similar issues, **longstanding sugar preference**	—
Triggers	Tobacco use, boredom, sugar-related cues, emotional deprivation, anxiety/mood fluctuations	Q3, Q21

Panel A summarizes the seven conceptual domains emerging from qualitative interviews. Panel B presents illustrative participant-derived concepts for each domain together with representative SuMQ items. The concepts guided item generation for the initial 25-item version of the SuMQ. Codes shown in bold were endorsed by at least nine participants (CVR > 0.64) and were considered essential during questionnaire development.

The original version of the SuMQ was developed in French. To enable international dissemination and facilitate use in non-French-speaking contexts, an English version of the questionnaire was produced using a forward–backward translation procedure. First, the French items were independently translated into English by a bilingual researcher with expertise in addiction and eating behavior. A backward translation into French was then performed by an independent bilingual translator who was not involved in the initial development of the scale. Discrepancies between the original and back-translated versions were reviewed by the research team and resolved by consensus to ensure conceptual equivalence rather than literal correspondence. The final English version is provided for research and screening purposes.

The SuMQ was administered to a general population sample recruited via social media. Inclusion criteria were: age ≥18 years, fluency in French, and consent to participate. Participants received written information detailing the study purpose and examples of high-sugar foods. They were asked a preliminary question: How would you rate your attraction to sugar-rich products? (5-point Likert scale: very low to very high = 0 to 4 points). Then, they completed the SuMQ using the same 5-point Likert scale (scored 0 to 4). Only age and gender were also collected.

### Statistical analysis

Item functioning was evaluated using the Partial Credit Rasch Model (27) for polytomous items (28). Rasch modeling was used to examine whether items contributed to a common underlying continuum of sugar-related difficulties and to identify items showing misfit, redundancy, or limited discrimination. Item fit was assessed using infit and outfit mean square statistics (29, 30). Values close to 1 were interpreted as indicating adequate fit to model expectations; values substantially greater than 1 were interpreted as underfit, reflecting unmodeled noise or unpredictable response patterns; and values substantially lower than 1 were interpreted as overfit, reflecting overly predictable or potentially redundant responses. Based on established Rasch recommendations and reporting guidelines (31, 32), items with mean square values >1.20 or <0.70, significant standardized residuals, low discrimination, or evidence of redundancy were considered candidates for removal. Overall model fit and item parameter invariance across score groups were additionally examined using Andersen’s likelihood ratio test (33). Rasch analyses were performed using the eRm package in R (34).

We used principal component analysis (PCA) and confirmatory factor analysis (CFA). PCA served as a data-reduction technique, transforming a large set of correlated variables into a smaller number of uncorrelated components that capture the maximum possible variance. CFA provided rigorous empirical evidence that the proposed theoretical model accurately represents the latent dimensions underlying the data. Model fit was evaluated using the comparative fit index (CFI), the Root Mean Square Error of Approximation (RMSEA), and its 90% confidence interval. CFA was performed using the Lavaan package for R (release 0.6.15). The sequential use of Rasch modeling, exploratory dimensionality analyses, and CFA was deliberately chosen to integrate item-level evaluation with assessment of the latent structure of the scale at different stages of its development.

Cronbach’s alpha estimates the reliability of a scale by quantifying the extent to which the items consistently measure the same underlying construct. We used the package PSYCH for R (release 2.5.6).

Receiver Operating Characteristic (ROC) analyses were conducted to determine the score that optimally balanced sensitivity and specificity. Participants reporting strong or very strong SuM were coded “1,” and those with none to moderate SuM were coded “0” (i.e., control group). The Area Under the Curve (AUC) and 95% confidence intervals (CIs) were computed. Bootstrap resampling (2,000 replicates) was used to estimate CIs for sensitivity and specificity. Thresholds were optimized using Youden’s J index (J = sensitivity + specificity – 1). We used Chi-square tests to assess whether self-reported “dependence” aligned with scores above the established cut-off, thereby examining concordance between SuMQ scores and self-reported severity.

The use of self-reported severity of SuM as the reference criterion for ROC analyses was not intended to serve as a diagnostic gold standard, but rather as a pragmatic anchoring variable to facilitate preliminary threshold estimation in the absence of an established clinical diagnosis for SuM. This approach is consistent with early-stage instrument development, in which cut-off scores are explored to support screening and research purposes rather than definitive clinical classification.

## Results

### Development of a questionnaire to define the misuse of sugar-rich products

#### Qualitative study for identifying keywords and core concepts

Thirteen individuals who self-identified as being highly “addicted” or “dependent” on sugar-rich products, and who did not report any specific attraction to other food categories (e.g., fatty or salty foods), were included in the qualitative study. Data saturation was assessed by three reviewers and achieved with 11 participants; two additional interviews were included but did not yield new keywords or concepts. The mean age of participants was 41.8 ± 15.4 years, with a women-to-men ratio of 80 to 20%. A total of 83 keywords or concepts were identified. Initially, 60 potential keywords were present in the interview guide; therefore, 23 new terms (38%) were introduced by participants.

#### Verbatim analysis, selection of key responses, and drafting of questionnaire items

Due to the high number of identified concepts, the most frequently cited were selected for inclusion. Fifteen concepts were present in over 90% of participants’ responses, and 29 in greater than 80%. The latter cut-off was chosen. Of these, four were considered redundant by the expert panel and were removed. The final list of 25 unique concepts was used to construct 25 questions. Where appropriate, the redaction of the questions was adapted from validated questionnaires such as the DSM-5 and YFAS-2.0. All questions were formatted using a 5-point Likert scale to allow quantitative evaluation.

The final questionnaire was reviewed and refined based on feedback from addiction specialists, diabetologists, and patients living with type 2 diabetes: the final French version is reported in Table 3 and the English version in Table 4.

**Table 3:** French version of the SuMQ.

Items	Les questions correspondent à votre comportement dans les 12 derniers mois	Jamais	1 fois par mois ou moins	2 à 4 fois par mois	2 à 3 fois par semaine	4 fois par semaine ou plus
1	La consommation de produits sucrés a-t-elle représenté un moment de plaisir intense?	0	1	2	3	4
2	La consommation de produits sucrés vous a-t-elle permis d’améliorer votre moral?	0	1	2	3	4
3	La consommation de produits sucrés vous a-t-elle servi à éviter des épisodes d’anxiété ou d’agitation?	0	1	2	3	4
4	La consommation de produits sucrés a-t-elle été une source importante de satisfaction dans votre quotidien?	0	1	2	3	4
5	Avez-vous ressenti des envies intenses et/ou urgentes à consommer des produits sucrés?	0	1	2	3	4
6	Votre consommation de produits sucrés a-t-elle été pour vous un véritable besoin plus qu’une simple envie?	0	1	2	3	4
7	Vous est-il arrivé de consommer des produits sucrés en dehors des repas?	0	1	2	3	4
8	Vous est-il arrivé de consommer des produits sucrés en quantité plus importante ou pendant une période plus prolongée que prévue?	0	1	2	3	4
9	Vous est-il arrivé de continuer à consommer des produits sucrés alors que vous n’aviez plus faim?	0	1	2	3	4
10	Vous est-il arrivé de continuer de consommer des produits sucrés alors même que leur goût ne vous attirait plus?	0	1	2	3	4
11	Vous est-il arrivé de consommer en cachette des produits sucrés pour éviter des remarques de votre entourage?	0	1	2	3	4
12	Vous est-il arrivé d’éviter d’avoir à disposition des produits sucrés par crainte de ne pouvoir contrôler des envies violentes?	0	1	2	3	4
13	La consommation de produits sucrés peut-elle provoquer chez vous l’envie d’en consommer encore plus?	0	1	2	3	4
14	En l’absence de produits sucrés, vous est-il arrivé de ressentir des symptômes de manque tels que: irritabilité, nervosité, agitation, anxiété, symptômes physiques?	0	1	2	3	4
15	Vous est-il arrivé de consommer des produits sucrés pour soulager ou éviter des symptômes de manque tels que: irritabilité, nervosité, agitation, anxiété, symptômes physiques?	0	1	2	3	4
16	Votre entourage vous a-t-il fait des remarques au sujet de votre consommation de produits sucrés?	0	1	2	3	4
17	Vous est-il arrivé de vous cacher à l’abri du regard des autres pour consommer des produits sucrés?	0	1	2	3	4
18	Avez-vous ressenti des envies incontrôlables d’acheter des produits sucrés?	0	1	2	3	4
19	La consommation de produits sucrés a-t-elle provoqué chez vous un sentiment de culpabilité?	0	1	2	3	4
20	Votre consommation de produits sucrés est-elle survenue sur des plages horaires spécifiques?	0	1	2	3	4
21	Vous est-il arrivé de consommer des produits sucrés pour traiter une anxiété, un épisode de stress ou une humeur dépressive?	0	1	2	3	4
22	Avez-vous ressenti la nécessité d’avoir des réserves de produits sucrés à votre disposition?	0	1	2	3	4
23	Avez-vous ressenti la nécessité de sortir à n’importe quelle heure et/ou de faire une grande distance pour aller acheter des produits sucrés parce que vous n’en aviez pas à disposition?	0	1	2	3	4
24	La consommation de produits sucrés a-t-elle provoqué chez vous un sentiment de souffrance?	0	1	2	3	4
25	Avez-vous continué à consommer des produits sucrés malgré l’existence de conséquences psychologiques ou physiques persistantes ou récurrentes susceptibles d’avoir été causées ou exacerbées par ces produits	0	1	2	3	4

It is the initial 25-item version. The questions in grey correspond to Q11. Items are rated on a 5-point Likert scale ranging from 0 (“Never”) to 4 (“4 times a week or more”). The original version of the questionnaire was developed in French.

**Table 4:** English version of the SuMQ: first version with 25 questions.

Items	The following questions refer to your behavior over the past 12 months	Never	Once a month or less	2–4 times a month	2–3 times a week	4 times a week or more
1	Did the consumption of sugar-rich products represent a moment of intense pleasure for you?	0	1	2	3	4
2	Did consuming sugar-rich products help improve your mood?	0	1	2	3	4
3	Did you use sugar-rich products to avoid episodes of anxiety or agitation?	0	1	2	3	4
4	Was the consumption of sugar-rich products an important source of satisfaction in your daily life?	0	1	2	3	4
5	Did you experience intense and/or urgent urges to consume sugar-rich products?	0	1	2	3	4
6	Did your consumption of sugar-rich products feel like a real need rather than a simple desire?	0	1	2	3	4
7	Did you consume sugar-rich products outside of meals?	0	1	2	3	4
8	Did you consume sugar-rich products in larger amounts or over a longer period than you had intended?	0	1	2	3	4
9	Did you continue to consume sugar-rich products even when you were no longer hungry?	0	1	2	3	4
10	Did you continue to consume sugar-rich products even when their taste was no longer appealing to you?	0	1	2	3	4
11	Did you consume sugar-rich products in secret to avoid comments from people around you?	0	1	2	3	4
12	Did you ever avoid having sugar-rich products available because you were afraid of not being able to control strong urges?	0	1	2	3	4
13	Did consuming sugar-rich products trigger the desire to consume even more?	0	1	2	3	4
14	In the absence of sugar-rich products, did you experience symptoms such as irritability, nervousness, agitation, anxiety, or physical discomfort?	0	1	2	3	4
15	Did you consume sugar-rich products to relieve or avoid symptoms such as irritability, nervousness, agitation, anxiety, or physical discomfort?	0	1	2	3	4
16	Did people around you make comments about your consumption of sugar-rich products?	0	1	2	3	4
17	Did you hide from the sight of others in order to consume sugar-rich products?	0	1	2	3	4
18	Did you experience uncontrollable urges to buy sugar-rich products?	0	1	2	3	4
19	Did the consumption of sugar-rich products lead to feelings of guilt?	0	1	2	3	4
20	Did your consumption of sugar-rich products occur at specific times of the day?	0	1	2	3	4
21	Did you consume sugar-rich products to cope with anxiety, stress, or a depressed mood?	0	1	2	3	4
22	Did you feel the need to keep sugar-rich products available or stocked?	0	1	2	3	4
23	Did you feel the need to go out at any time and/or travel a long distance to buy sugar-rich products because none were available?	0	1	2	3	4
24	Did the consumption of sugar-rich products cause you feelings of distress or suffering?	0	1	2	3	4
25	Did you continue to consume sugar-rich products despite persistent or recurrent psychological or physical consequences that may have been caused or worsened by their consumption?	0	1	2	3	4

The questions in grey correspond to Q11. Items are rated on a 5-point Likert scale ranging from 0 (“Never”) to 4 (“4 times a week or more”). The original version of the questionnaire was developed in French.

### Quantitative inferential validation

Five hundred and forty-nine participants were recruited, including 485 women. The mean age was 38 years (S. D. = 10.42). The mean weight was 72 kg (S. D. = 17.32), and the Body mass index values ranged from 17.89 to 46.06 (mean = 25.01, S. D. = 4.91). They were recruited through social networks and groups for patients or relatives of patients with metabolic disorders, eating disorders, or obesity.

Given concerns about data integrity in online research, we conducted *post hoc* quality checks, including screening for duplicate records, detecting uniform response patterns, and assessing coherence across self-reported variables. No evidence of systematic, automated, or large-scale non-authentic responding was observed. Sensitivity analyses excluding uniform responders and duplicate response profiles did not materially alter the psychometric results.

The Andersen’s likelihood ratio test based on the initial Q25 questionnaire failed to support model consistency and item parameter invariance across score groups (χ^2^ = 64.61, df = 27, *p* < 0.001). Examination of individual item-fit statistics identified several items with evidence of misfit or limited contribution to the measurement continuum. Items 2, 10, 12, 20, and 23 showed underfit, as indicated by elevated outfit and/or infit mean square values and significant item-level fit statistics. Item 20 showed the strongest misfit pattern, with both outfit and infit mean square values exceeding the predefined threshold. Item 23 also showed elevated outfit statistics and the lowest discrimination value. Other items were considered for removal due to redundancy, lower discriminative power, or conceptual overlap with retained items. The exclusion of these questionable items resulted in a substantial improvement in Andersen’s test (χ^2^ = 32.8, df = 19, *p* = 0.026), justifying a first reduction to the Q18 questionnaire (Table 5).

**Table 5:** Rasch item-fit statistics for the initial 25-item version of the SuMQ.

Item	χ^2^	df	*p*	Outfit MSQ	Infit MSQ	Outfit *t*	Infit *t*	Discrimination
Q1	548.575	546	0.461	1.003	0.991	0.067	−0.130	0.650
**Q2**	**695.284**	**546**	**0.000**	**1.271**	**1.151**	**3.265**	**2.438**	**0.626**
Q3	575.362	546	0.186	1.052	1.051	0.479	0.796	0.673
Q4	549.650	546	0.448	1.005	1.029	0.092	0.510	0.673
Q5	385.969	546	1.000	0.706	0.718	−4.784	−5.254	0.791
Q6	428.647	546	1.000	0.784	0.889	−2.306	−1.771	0.736
Q7	592.746	546	0.081	1.084	1.032	1.367	0.589	0.625
Q8	448.952	546	0.999	0.821	0.895	−2.720	−1.726	0.711
Q9	535.177	546	0.622	0.978	0.975	−0.329	−0.405	0.690
**Q10**	**630.858**	**546**	**0.007**	**1.153**	**1.096**	**0.937**	**1.083**	**0.571**
Q11	442.288	546	1.000	0.809	0.926	−1.330	−0.901	0.686
**Q12**	**627.520**	**546**	**0.009**	**1.147**	**1.168**	**1.151**	**2.253**	**0.629**
Q13	432.947	546	1.000	0.791	0.844	−2.819	−2.645	0.753
**Q14**	**363.199**	**546**	**1.000**	**0.664**	**0.901**	**−2.101**	**−1.147**	**0.710**
Q15	436.098	546	1.000	0.797	0.875	−1.569	−1.714	0.726
Q16	534.001	546	0.635	0.976	1.110	−0.130	1.270	0.612
Q17	388.658	546	1.000	0.711	0.791	−1.939	−2.467	0.718
Q18	397.202	546	1.000	0.726	0.794	−3.325	−3.077	0.745
Q19	461.016	546	0.997	0.843	0.924	−1.928	−1.235	0.739
**Q20**	**731.609**	**546**	**0.000**	**1.337**	**1.324**	**2.763**	**4.549**	**0.583**
Q21	410.741	546	1.000	0.751	0.864	−2.731	−2.096	0.741
Q22	524.738	546	0.736	0.959	1.033	−0.259	0.474	0.674
**Q23**	**736.580**	**546**	**0.000**	**1.347**	**1.158**	**1.407**	**1.166**	**0.451**
Q24	496.600	546	0.936	0.908	1.022	−0.402	0.272	0.642
Q25	480.991	546	0.979	0.879	0.961	−0.892	−0.531	0.706

Outfit and infit mean square (MSQ) values close to 1 indicate a good fit to the Rasch model. Values >1.20 or <0.70 were considered potential indicators of item misfit or redundancy and were used to guide item reduction. Candidate items are highlighted in bold.

Principal component analysis of the Q18 increased the proportion of variance explained by the first component to 53%, and exploratory factor analyses suggested a predominantly unidimensional structure. However, items 11, 16, 17, and 24 showed weaker associations with the main dimension (Table 6). Internal consistency remained very high (Cronbach’s *α* = 0.947, 95% CI: 0.939–0.954), suggesting potential redundancy among some retained items.

**Table 6:** Exploratory factor loadings for the initial (Q25) and reduced (Q18) versions of the SuMQ.

Panel A. Initial 25-item version (Q25)			
Item	Factor 1	Factor 2	Factor 3
Q4	**0.76**	0.15	0.20
Q1	**0.73**	–	0.19
Q2	**0.69**	0.21	–
Q7	**0.64**	0.23	–
Q6	**0.64**	0.37	0.21
Q5	**0.62**	0.43	0.27
Q9	**0.60**	0.26	0.28
Q3	**0.56**	0.37	0.20
Q8	**0.56**	0.35	0.29
Q13	**0.54**	0.41	0.32
Q20	**0.51**	0.23	0.25
Q19	**0.51**	0.35	0.41
Q25	**0.46**	0.40	0.36
Q14	0.23	**0.79**	0.26
Q15	0.29	**0.77**	0.22
Q18	0.35	**0.64**	0.32
Q21	0.40	**0.59**	0.28
Q22	0.43	**0.47**	0.26
Q23	–	**0.44**	0.27
Q12	0.34	**0.42**	0.34
Q16	0.30	**0.41**	0.40
Q17	0.21	0.36	**0.84**
Q11	0.27	0.28	**0.77**
Q24	0.31	0.38	**0.47**
Q10	0.30	0.35	**0.38**

Panel B. Reduced 18-item version (Q18)		
Item	Factor 1	Factor 2
Q5	**0.75**	0.34
Q6	**0.73**	0.28
Q4	**0.65**	0.23
Q1	**0.64**	0.21
Q13	**0.64**	0.39
Q8	**0.64**	0.33
Q9	**0.62**	0.31
Q3	**0.62**	0.30
Q21	**0.60**	0.42
Q18	**0.59**	0.46
Q19	**0.58**	0.45
Q15	**0.58**	0.43
Q25	**0.56**	0.43
Q14	**0.55**	0.46
Q17	0.25	**0.92**
Q11	0.30	**0.79**
Q24	0.42	**0.53**
Q16	0.41	**0.49**

Only loadings ≥0.15 are displayed. Primary loadings are shown in bold. Items are grouped according to their dominant factor. Panel A. Initial 25-item version (Q25), Panel B. Reduced 18-item version (Q18).

Hierarchical clustering of item correlations was therefore performed to identify highly correlated item pairs and clusters that could reflect redundancy. Several strongly correlated item pairs were identified, including items (8, 9), (5, 6), (1, 4), (3, 21), (14, 15), (19, 24), and (11, 17) (Table 7). Within each pair, the item showing the lower contribution to measurement performance or greater conceptual overlap was removed, resulting in the final 11-item version (Q11).

**Table 7:** Correlations between total scores of the Q11, Q18, and Q25 versions of the SuMQ and self-reported severe SuM.

Items	Pred11	Pred18	Pred25	Severe misuse
Pred11	1.000	0.986	0.977	0.634
Pred18	0.986	1.000	0.991	0.623
Pred25	0.977	0.991	1.000	0.629
Severe misuse	0.634	0.623	0.623	1.000

**Figure 2:**
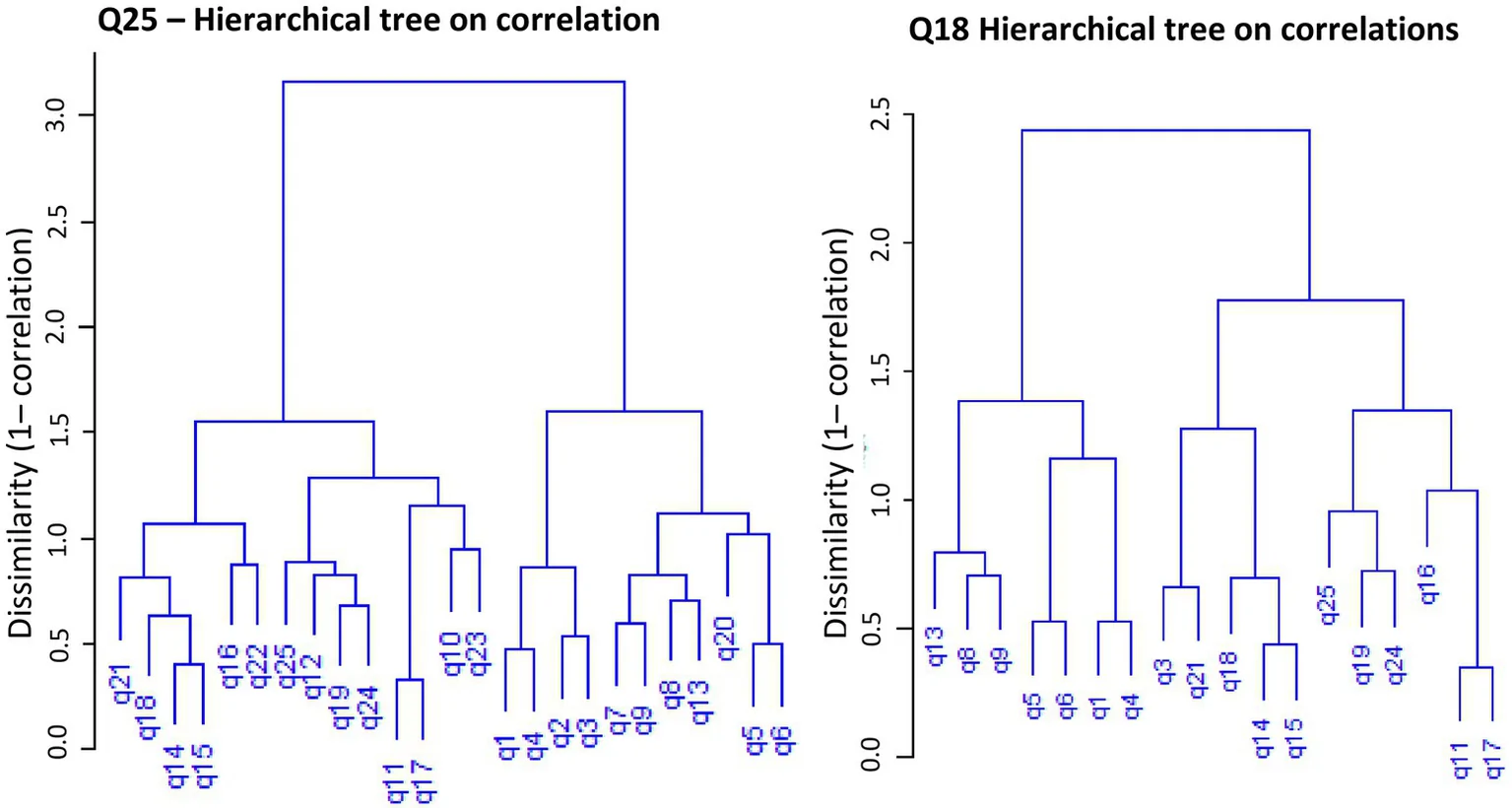
Hierarchical clustering of item correlations used to identify potentially redundant items during questionnaire reduction.

Q11, composed of 1, 3, 5, 8, 11, 13, 14, 16, 18, 19, and 25 items, was characterized by α = 0.915 [0.901, 0.923] and the first PCA component with 55% of the explained variance. A CFA provided factor loadings (standardized regression coefficients) above 0.40 (range 0.63–0.80). The fit indices indicated an acceptable model fit (CFI = 0.96; robust RMSEA = 0.07, 90% CI [0.06, 0.09]). Nevertheless, caution is warranted when interpreting model fit, as the upper bound of the RMSEA confidence interval falls within the range associated with mediocre fit in the psychometric literature. The determination/discrimination of the Q11, Q18, and Q25 (Figure 2) provides evidence of the overall superiority of the Q11 short form.

A detailed administration, scoring, and interpretation guide for the validated SuMQ is provided in Supplementary Appendix S1A. The original developmental 25-item version is provided in Supplementary Appendix S1B.

### Exploratory threshold estimation

**Figure 3:**
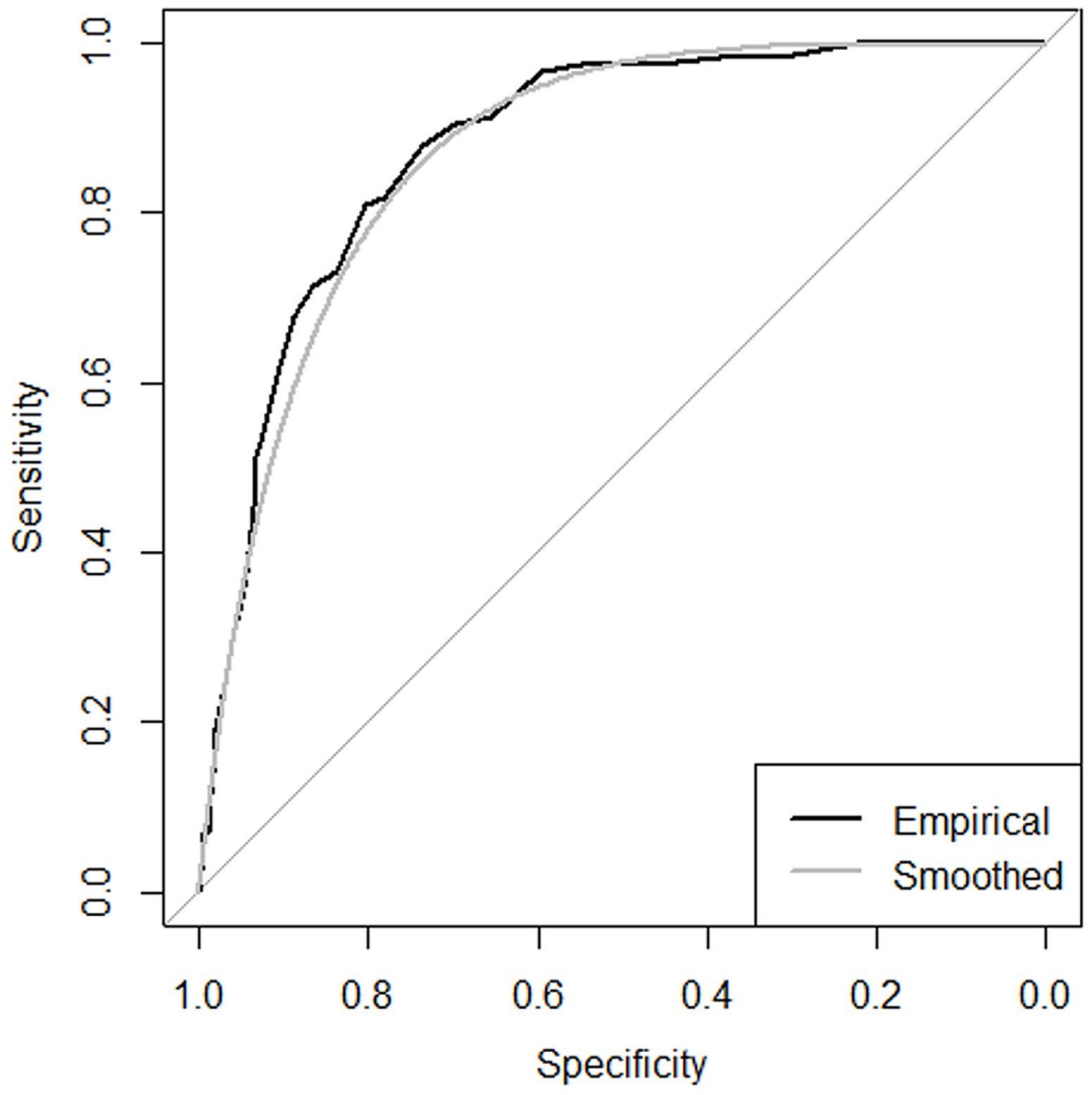
Receiver operating characteristic (ROC) curve evaluating the ability of the final SuMQ score to discriminate participants reporting severe SuM from those reporting absent, mild, or moderate SuM. The area under the curve (AUC) is indicated as a measure of discriminative performance.

**Figure 4:**
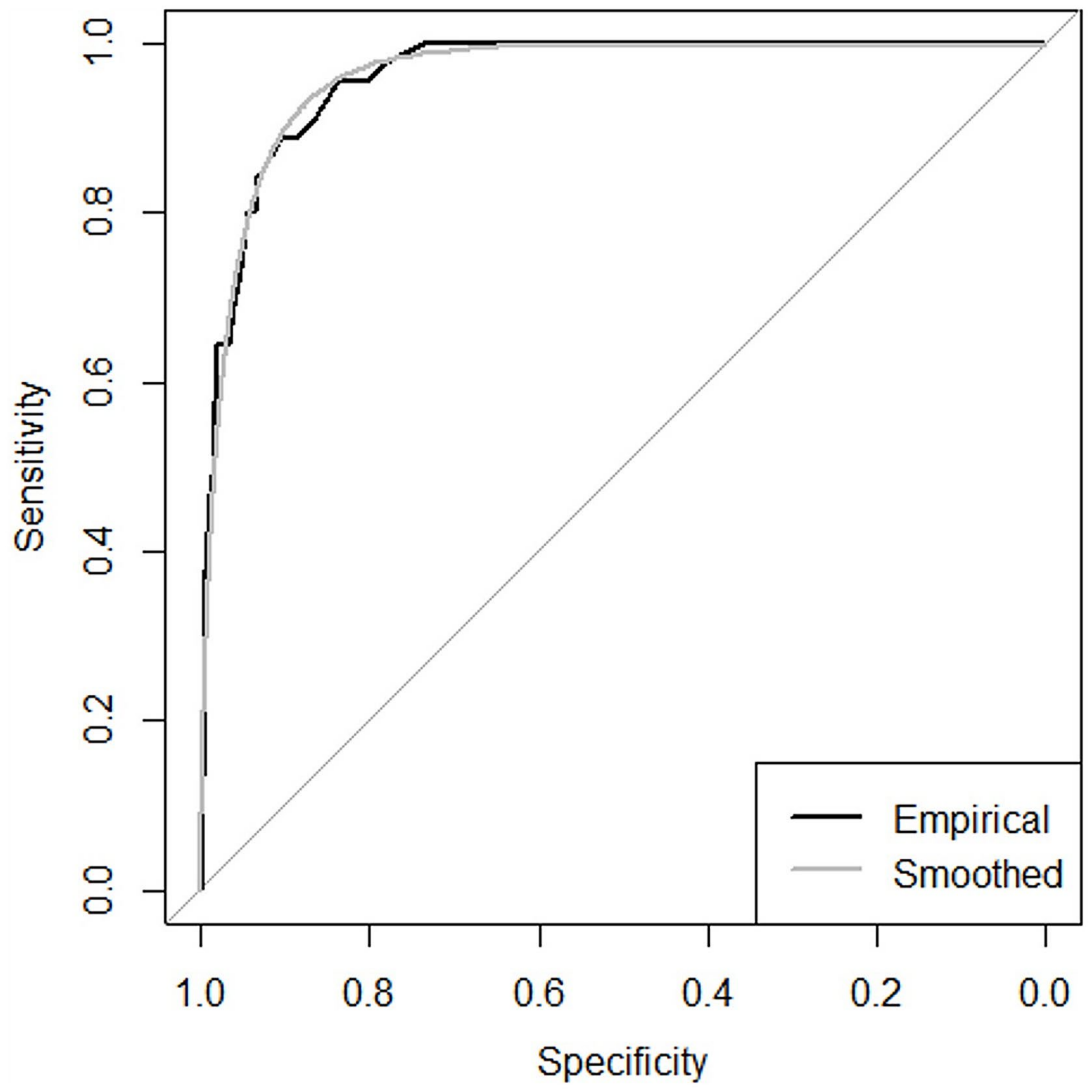
Receiver operating characteristic (ROC) curve evaluating the ability of the final SuMQ score to discriminate participants reporting very severe SuM from all other participants (ROC = 0.96). The area under the curve (AUC) is used as a measure of discriminative performance.

To determine cut-off scores, ROC curve analysis was performed. For distinguishing high SuM from controls, the area under the curve (AUC) was 0.88 (95% CI: 0.85–0.91) (Figure 3). A score ≥14 balanced sensitivity (Se = 0.81, 95% CI: 0.74–0.88) and specificity (Sp = 0.80, 95% CI: 0.77–0.84), with a Youden Index of 1.61. For very high SuM, the AUC was 0.96 (95% CI: 0.94–0.98) (Figure 4). A score ≥18 achieved Se = 0.89 (95% CI: 0.80–0.98) and Sp = 0.90 (95% CI: 0.87–0.93), with a Youden index of 1.79.

## Discussion

The present study introduces SuM as a measurable behavioral phenotype and provides the first psychometrically evaluated instrument specifically developed to operationalize this construct. Using a sequential mixed-methods approach grounded in patients’ lived experiences, we identified the principal experiential dimensions of SuM. We developed a concise 11-item questionnaire demonstrating strong internal consistency and a stable unidimensional structure.

Excessive consumption of sweet products is a global health problem whose prevalence is steadily increasing. According to the severity of the potential complications of this overconsumption, identifying maladaptive patterns of sugar-rich food consumption represents an important public health challenge. Early detection of SuM is clinically important, especially for individuals with hyperglycemia, since it could prevent the progression to diabetes (35). High sugar intake is also linked to cardiovascular disease (36) and multiple cancers (37, 38) independently of obesity, suggesting potential direct adverse effects. For these latter complications, early detection of SuM could also lead to preventive intervention.

On the other hand, determining the amounts of sweet products consumed is not sufficient. It is equally important to understand consumption patterns and identify behaviors associated with the risk of Sugar Misuse. Without this information, advice and interventions aimed at reducing excessive consumption may be less effective.

Although several dimensions identified by the SuMQ overlap with constructs commonly described in addictive disorders (e.g., craving, loss of control, persistence despite negative consequences), the present study does not assume the existence of a formal sugar addiction nor does it aim to establish a diagnostic entity. Instead, SuM is conceptualized here as a maladaptive behavioral pattern situated along a continuum of eating behaviors, which may share phenomenological similarities with addiction without implying equivalence or diagnostic validity. This distinction is particularly important given ongoing debates regarding the applicability of SUD frameworks to eating behaviors and aligns with perspectives emphasizing addictive-like eating behaviors rather than categorical diagnoses.

The SuMQ should not be interpreted as evidence for a distinct nosographic entity. Instead, it may contribute to ongoing discussions regarding the boundaries between addictive-like eating, emotional eating, and binge-eating behaviors. By focusing specifically on sugar-rich products and grounding item development in patient-reported experiences, the present study adopts a phenomenological and dimensional approach consistent with current shifts away from rigid categorical models in eating disorder research.

The present findings should also be interpreted in light of ongoing debates regarding food addiction and UPFUD. Although several phenomenological features assessed by the SuMQ, such as craving, loss of control, and persistence despite adverse consequences, overlap with dimensions commonly discussed in these frameworks, the present study was not designed to determine whether SuM constitutes a subtype of food addiction or UPFUD. Rather, the SuMQ aims to quantify a specific pattern of problematic sugar-related experiences and behaviors observed in clinical settings.

Accordingly, the SuMQ should not be interpreted as evidence supporting the existence of a distinct addictive disorder related to sugar. Instead, it provides a standardized instrument for assessing sugar-related difficulties, regardless of the theoretical framework used to explain them. Importantly, the absence of concurrent YFAS-2.0 assessment in the present study precluded direct evaluation of convergent and discriminant validity with established food addiction measures. Future studies should therefore examine the relationships among SuMQ scores, YFAS-2.0 measures, binge-eating and craving-related scales, and emerging UPFUD assessment tools to better characterize areas of convergence and divergence among these constructs.

A methodological limitation of the present study is the use of Rasch modeling, exploratory dimensionality analyses, and CFA within a single sample. While this stepwise approach was implemented to ensure internal coherence of the scale, first at the item level (Rasch model), then at the dimensional level (PCA), and finally at the latent structure level (CFA), the absence of an independent validation sample may have increased the risk of overfitting. Although Rasch modeling provides strong constraints on item functioning, future studies should replicate the factor structure and model fit of the SuMQ in independent samples to confirm the stability and generalizability of the proposed structure. Such replication will be essential to determine whether the observed dimensional structure reflects stable properties of SuM or sample-specific characteristics.

A crucial goal is to define a SuM, which is a behavior widely accepted in the general population and among many practitioners. This concept, however, is not currently recognized, and therefore defining it, or at least developing a questionnaire to identify it, requires several precautions for each step.

The first step was to conduct a qualitative study in order to understand the phenomenon we sought to describe. The development of an interview guide was primarily based on patient statements describing a strong, specific craving for sugar in a previous study (13). To make the results more reliable, two additional interviews were included after data saturation (without adding any additional keywords confirming data saturation). Keywords were selected during a parallel reading of the verbatim by three clinicians (two MDs and one psychologist). The interview guide was enriched by 38% more keywords through participant input, revealing additional, unexpected themes such as stress-related consumption, guilt, social withdrawal, and storage of sweet products.

To define a homogeneous clinical profile, we intentionally selected participants reporting the most severe sugar-related difficulties. Therefore, only the subjects defining themselves as “highly addicted” or having a “severe addiction” participated in the qualitative study. Most importantly, it was essential to include only participants who specifically reported a strong preference for sweet products without marked attraction to fatty or salty foods. Thus, the SuMQ was designed to assess misuse of sugar-rich products rather than a general eating disorder. For the development of the questionnaire, we also wanted to select criteria corresponding to a clear clinical entity; so, only keywords described by at least 80% of the participants were retained. The 25 most prevalent items were selected to compose the first version of the SuMQ, which captures various dimensions of SuM: behavioral characteristics, consequences, triggers, difficulty in behavior change, craving, and loss of control. This first version has been read and corrected by three MDs (two in addictology and one in diabetology), as well as by diabetic patients, to ensure that the questions are clear and easy to understand. Where possible, the drafting of questions was inspired by the already validated questionnaires, DSM-5, and YFAS-2.0. The Q25 was assessed in the general population by recruiting volunteers via social networks, including participants with varying degrees of SuM, ranging from none to very severe. To eliminate duplicate questions while keeping the same psychometric quality of SuMQ, we used the Rasch model. This allowed us to obtain a version with 11-item version (Q11), that is easy to administer in routine clinical practice, with higher discrimination than Q25 and having a very good internal consistency. Finally, specific cutoffs for the Q11 were evaluated using self-assessments that described severe or very severe SuM. The two scores obtained were relatively close (14 and 18/44), and we considered this threshold clinically meaningful to identify severe SuM given its potential risks. A score ≥14/44 was identified as a preliminary threshold associated with self-reported severe SuM. It may therefore help identify individuals who could benefit from further assessment in research or clinical contexts.

Strengths and weaknesses. Two potential limitations should be acknowledged. First, the inability to exclude participants with severe psychiatric or neurological conditions who responded to the online questionnaire. However, the large number of respondents allowed limiting the possible impact of some outlier responses. Second, the sex ratio of volunteers was strongly in favor of women, which could represent a bias. In the future, using the questionnaire on a larger scale will make it possible to compare any differences between men and women.

An additional limitation concerns the characteristics of the validation sample. Participants were recruited through social networks and groups involving individuals with metabolic disorders, obesity, or eating-related concerns. Consequently, the sample may have been enriched in individuals presenting elevated levels of problematic eating behaviors and may not be fully representative of the general population. Replication of the psychometric properties and proposed cut-off values in broader community-based samples will therefore be necessary.

Several limitations should be acknowledged regarding the external validation of the SuMQ. The absence of concurrent administration of the YFAS-2.0 prevented direct assessment of convergent and discriminant validity with established food addiction measures. In the present study, preliminary validation efforts primarily focused on internal structure, item functioning, and threshold estimation, while convergent and discriminant validity with established measures of eating behavior or addictive-like eating were not assessed. This choice reflects the exploratory nature of this first preliminary validation step, which aimed to ground the instrument in patient-reported experiences and psychometric coherence rather than in predefined theoretical frameworks. As a consequence, the extent to which SuMQ scores overlap with or diverge from existing constructs such as food addiction, binge-eating, or emotional eating remains to be formally examined.

Future studies should therefore evaluate the convergent validity of the SuMQ with instruments such as the Yale Food Addiction Scale, binge-eating measures, or craving-related scales, as well as its discriminant validity with adjacent but distinct constructs. Such analyses will be essential to clarify the specific contribution of the SuMQ and to determine whether SuM captures a partially overlapping or distinct behavioral phenotype.

The identification of preliminary cut-off scores should be interpreted with caution. Accordingly, these thresholds should be interpreted as provisional research thresholds rather than clinically validated diagnostic cut-offs.

In the absence of an established diagnostic framework for SuM, the proposed thresholds are not intended to support clinical diagnosis or treatment decisions. Rather, they should be viewed as hypothesis-generating markers that may help flag individuals who report particularly severe difficulties with sugar-rich food consumption and who might benefit from further clinical evaluation or tailored behavioral support. Future studies will be required to determine whether these thresholds are stable across populations, predictive of health-related outcomes, or useful for monitoring change over time.

Although an English version of the SuMQ is provided, the present study did not aim to formally assess cross-cultural validity or measurement invariance across languages. Further studies will therefore be required to evaluate the psychometric properties of the English version in independent samples and to examine its equivalence with the original French version.

While the concept of sugar addiction remains debated, clinically observed sugar-focused maladaptive consumption patterns warrant careful behavioral characterization without presupposing diagnostic status. While the concept of sugar addiction remains debated, a substantial body of experimental and clinical literature continues to investigate sugar-related addictive-like behaviors (39, 40). Moreover, addictive-like behaviors provoked by some human foods like SSBs and chocolate have been described for many years (41, 42), and a questionnaire with preliminary psychometric validation, such as the SuMQ, is a necessary step toward a better understanding of sugar-related behaviors and the evaluation of therapeutic strategies (43). Finally, it seems that in some areas of health, clinical understanding progresses faster than theoretical concepts. Thus, modalities for addressing food addiction are starting to be published even though the very existence of this entity remains a controversial issue (44).

To conclude, the present study provides the first preliminary psychometric validation of the SuMQ, a standardized instrument specifically developed to operationalize SuM as a dimensional behavioral construct. By providing a reproducible measure grounded in patients’ lived experiences, the SuMQ offers a new tool for clinical research, epidemiological studies, and future intervention trials investigating maladaptive sugar-rich food consumption.

## Data Availability

The raw data supporting the conclusions of this article will be made available by the authors, without undue reservation.
