# Supplementary material for: Assessing maladaptive sugar-rich food consumption: development and preliminary validation of the sugar misuse questionnaire
Source: Front Nutr. 2026 Aug 7;13:1855019. doi: 10.3389/fnut.2026.1855019 (PMC13495693; doi:10.3389/fnut.2026.1855019)
Supplement: Supplementary file 1 [file Data_Sheet_1.zip › Supplementary informations.docx]

**Supplementary informations**

**Materials and methods**

**Qualitative development of the SuMQ**

Patients from addiction and diabetology units who self-identified as being “addicted to” or having a strong craving for sugar-rich foods were invited to participate in interviews exploring their potential sugar misuse (SuM). Recruitment intentionally focused on individuals reporting the most severe forms of sugar-related difficulties to maximize the identification of clinically meaningful experiential dimensions rather than to estimate prevalence.

The interview guide was developed based on previous literature, including evidence showing increased sugar craving during alcohol withdrawal (Alarcon et al., 2020), and refined through expert review by specialists in addiction and eating disorders. Interviews were conducted by clinically trained researchers using a semi-structured format starting with open-ended questions followed by more focused prompts.

The guide covered nine thematic areas addressing daily sugar consumption patterns, subjective importance of sugar in participants’ lives, internal and external triggers of consumption, potential withdrawal-like experiences, and emotional or functional consequences. In total, the guide included 60 possible questions: 41 derived from clinical observations, 12 adapted from the Yale Food Addiction Scale (YFAS), and 7 from DSM-5 substance use disorder criteria.

Audio-recorded interviews were transcribed verbatim and analyzed using QDA Miner Lite (Provalis Research). Content analysis combined inductive and deductive approaches. Transcripts were coded line-by-line to identify concepts related to triggers, behavioral patterns, consequences, frequency, and severity of sugar misuse. When new concepts emerged, the coding scheme was iteratively updated.

Coding continued until thematic saturation was reached, defined as the absence of new conceptual categories in successive interviews. Two additional interviews were conducted after saturation to confirm stability of the coding structure.

Concept frequency was calculated as the proportion of participants mentioning each theme. Items were retained based on frequency and clinical relevance. Redundant concepts were merged, and the most representative item formulation was selected.

To identify essential items, the Content Validity Ratio (CVR) method (Lawshe, 1975) was applied using the formula:

CVR = (Ne − N/2) / (N/2)

where N is the total number of participants and Ne the number identifying the item as essential. Items with CVR ≥ 0.64 were retained.

Items were formulated using wording inspired by DSM-5 and YFAS constructs when relevant and organized into a preliminary questionnaire rated on a 5-point Likert scale. The instrument was reviewed by experts in addiction and diabetology as well as by patients to ensure clarity and content validity.

The original questionnaire was developed in French. An English version was generated using forward–backward translation by bilingual researchers to ensure conceptual equivalence.

**Statistics**

Item functioning was evaluated using the Partial Credit Rasch Model for polytomous items (Masters, 1982; Rasch, 1960). Rasch modeling was used to examine whether items contributed to a common underlying continuum of sugar-related difficulties and to identify items showing misfit, redundancy, or limited discrimination. Item fit was assessed using infit and outfit mean square statistics (Wright & Masters, 1982). Values close to 1 were interpreted as indicating adequate fit to model expectations; values substantially greater than 1 were interpreted as underfit, reflecting unmodeled noise or unpredictable response patterns; and values substantially lower than 1 were interpreted as overfit, reflecting overly predictable or potentially redundant responses. Based on established Rasch recommendations and reporting guidelines (Smith et al., 2003; Van de Winckel et al., 2022), items with mean square values >1.20 or <0.70, significant standardized residuals, low discrimination, or evidence of redundancy were considered candidates for removal. Overall model fit and item parameter invariance across score groups were additionally examined using Andersen’s likelihood ratio test (Andersen, 1973). Rasch analyses were performed using the eRm package in R (Mair & Hatzinger, 2007).

We used the Principal Component Analysis (PCA) as a data-reduction technique to transform a large set of correlated variables into a smaller number of uncorrelated components that capture the maximum possible variance. The Confirmatory Factorial Analysis (CFA) was used to provide rigorous empirical evidence that a proposed theoretical model accurately represents the latent dimensions underlying the data. The statistics used to evaluate model fit were the comparative fit index (CFI), the Root Mean Square Error of Approximation (RMSEA) and its 90% confidence interval. CFA was performed using the Lavaan package for R (release 0.6.15).

The sequential use of Rasch modeling, exploratory dimensionality analyses, and confirmatory factor analysis was deliberately chosen to combine item-level evaluation with assessment of the latent structure of the scale at different stages of its development.

The Cronbach’s alpha estimates the reliability of a scale by quantifying the extent to which the items consistently measure the same underlying construct. We used the package PSYCH for R (release 2.5.6)

Receiver Operating Characteristic ROC analyses were conducted to determine the score that optimally balanced sensitivity and specificity. Participants reporting strong or very strong SuM were coded “1”, and those with none to moderate SuM were coded “0” (i.e. control group). The Area Under the Curve (AUC) and 95% confidence intervals (CIs) were computed. Bootstrap resampling (2,000 replicates) was used to estimate CIs for sensitivity and specificity. Thresholds were optimized using Youden’s J index (J = sensitivity + specificity – 1). We used Chi-square tests to assess whether self-reported “dependence” aligned with scores above the established cut-off, thereby validating the questionnaire’s diagnostic accuracy.

The use of self-reported severity of SuM as the reference criterion for ROC analyses was not intended to serve as a diagnostic gold standard, but rather as a pragmatic anchoring variable to facilitate preliminary threshold estimation in the absence of an established clinical diagnosis for SuM. This approach is consistent with early-stage instrument development, in which cut-off scores are explored to support screening and research purposes rather than definitive clinical classification.

**RASCH results**

The Partial Credit Rasch Model (Masters, 1982) was used to evaluate item functioning and scale structure. Item fit was assessed using infit and outfit mean square statistics, with values close to 1 indicating adequate fit to model expectations. Values substantially greater than 1 were interpreted as underfit, reflecting unmodeled noise or unpredictable response patterns, whereas values substantially lower than 1 were interpreted as overfit, suggesting redundancy or overly predictable responses (Wright & Masters, 1982).

Item removal decisions were based on a combination of criteria including item misfit (infit or outfit mean square values >1.20), overfit suggesting redundancy (<0.70), significant standardized residuals, low discrimination coefficients, redundancy identified through hierarchical clustering analyses, and conceptual overlap with retained items. Global model fit and item parameter invariance across score groups were evaluated using Andersen’s likelihood ratio test (Andersen, 1973). A significant result was interpreted as evidence that the Rasch model assumptions were not fully satisfied.

Rasch analyses were conducted using the eRm package in R (Mair & Hatzinger, 2007). The item-person map and item characteristic curves were examined to assess targeting and category functioning.

**References**

Andersen EB. A goodness of fit test for the Rasch Model. Psychometrika 1973;38:123-40.

Bond TG, Fox CM. Applying the Rasch Model: Fundamental Measurement in the Human Sciences. 2nd ed. Mahwah, NJ: Lawrence Erlbaum Associates; 2007.

Mair P, Hatzinger R. (2007). Extended Rasch modeling: The eRm package for the application of IRT models in R. Journal of Statistical Software 2007;20(9):1-20.

Masters GN. A rasch model for partial credit scoring. Psychometrika 1982;47:149-174.

Rasch, G. (1960). Probabilistic models for some intelligence and attainment tests. University of Chicago Press.

Smith RM, Linacre JM, Smith EV Jr. Guidelines for manuscripts. Journal of Applied Measurement. 2003;4:198–204.

Van de Winckel A, Kozlowski AJ, Johnston MV, Weaver J, Grampurohit N, Terhorst L, Juengst S, Ehrlich-Jones L, Heinemann AW, Melvin J, Sood P, Mallinson T. Reporting Guideline for RULER: Rasch Reporting Guideline for Rehabilitation Research: Explanation and Elaboration. Arch Phys Med Rehabil. 2022;103(7):1487–1498.

Wright, B. & Masters, G. (1982). Rating scale analysis. Chicago, IL: MESA Press.

**Matrix Block**

Location Threshold 1 Threshold 2 Threshold 3 Threshold 4

q1 -1.12853	-2.72934	-1.25168	-0.51176	-0.02135

q2 -0.20900	-1.04638	-0.62391	0.08174	0.75257

q3	0.23277	0.02940	-0.50349	0.55963	0.84555

q4 -0.54274	-1.47813	-0.87015	0.06883	0.10848

q5 -0.26723	-1.56926	-0.31330	0.20199	0.61167

q6	0.08181	-0.41429	0.06275	0.04808	0.63069

q7 -1.55810	-3.66615	-2.09501	-0.20390	-0.26733

q8 -0.07766	-1.53827	-0.07066	0.82829	0.47001

q9 -0.50742	-2.19908	-0.66431	0.43724	0.39648

q10 0.91598	0.76142	0.58746	1.23779	1.07727

q11 0.77225	0.53826	0.43560	1.25091	0.86424

q12 0.31911	0.17022	-0.00746	0.99741	0.11628

q13 -0.06973	-1.00298	-0.27486	0.60620	0.39274

q14 0.68562	0.88237	0.29622	1.47160	0.09229

q15 0.49587	0.30623	0.26799	0.77708	0.63217

q16 0.71295	0.32601	0.82699	1.24986	0.44895

q17 0.92264	0.69406	0.96211	0.80945	1.22493

q18 0.52530	-0.58249	0.46264	1.53609	0.68496

q19 -0.20145	-0.91114	-0.20614	0.58744	-0.27594

q20 0.07212	-0.13325	-0.16236	0.16819	0.41589

q21 0.28863	-0.39671	0.17257	0.73154	0.64710

q22 0.39303	0.29729	0.18575	0.47817	0.61090

q23 1.43814	1.50635	1.57271	1.64779	1.02573

q24 0.71408	1.13597	0.37496	1.01367	0.33171

q25 0.30294	0.27376	0.06471	0.46128	0.41200

Plot Item Characteristic Curves ICC

Plot Person-Item Map

Item-Fit statistics

**Itemfit Statistics for Q18:**

Chisq df p-value Outfit MSQ Infit MSQ Outfit t Infit t Discrim

q1 572.527 533	0.115	1.072	1.043	1.147	0.746	0.621

q3 616.412 533	0.007	1.154	1.111	1.391	1.652	0.656

q4 579.697 533	0.079	1.086	1.103	1.244	1.693	0.647

q5 394.944 533	1.000	0.740	0.734	-4.296 -4.845	0.783

q6 453.321 533	0.995	0.849	0.942	-1.647 -0.873	0.720

q8 474.682 533	0.967	0.889	0.951	-1.686 -0.758	0.687

q9 583.043 533	0.066	1.092	1.054	1.463	0.910	0.657

q11 438.289 533	0.999	0.821	0.950	-1.315 -0.586	0.688

q13 467.263 533	0.981	0.875	0.898	-1.706 -1.662	0.734

q14 349.022 533	1.000	0.654	0.925	-2.307 -0.837	0.712

q15 444.245 533	0.998	0.832	0.911	-1.355 -1.178	0.722

q16 584.545 533	0.060	1.095	1.182	0.757	2.036	0.594

q17 387.897 533	1.000	0.726	0.821	-1.927 -2.070	0.721

q18 405.758 533	1.000	0.760	0.831	-3.049 -2.493	0.730

q19 471.906 533	0.973	0.884	0.957	-1.455 -0.660	0.731

q21 431.238 533	1.000	0.808	0.919	-2.177 -1.201	0.725

q24 557.389 533	0.225	1.044	1.075	0.289	0.821	0.632

q25 512.185 533	0.734	0.959	1.019	-0.284	0.282	0.693

### **Itemfit Statistics**

Chisq df p-value Outfit MSQ Infit MSQ Outfit t Infit t Discrim

q1 548.575 546	0.461	1.003	0.991	0.067 -0.130	0.650

q2 695.284 546	0.000	1.271	1.151	3.265	2.438	0.626

q3 575.362 546	0.186	1.052	1.051	0.479	0.796	0.673

q4 549.650 546	0.448	1.005	1.029	0.092	0.510	0.673

q5 385.969 546	1.000	0.706	0.718	-4.784 -5.254	0.791

q6 428.647 546	1.000	0.784	0.889	-2.306 -1.771	0.736

q7 592.746 546	0.081	1.084	1.032	1.367	0.589	0.625

q8 448.952 546	0.999	0.821	0.895	-2.720 -1.726	0.711

q9 535.177 546	0.622	0.978	0.975	-0.329 -0.405	0.690

q10 630.858 546	0.007	1.153	1.096	0.937	1.083	0.571

q11 442.288 546	1.000	0.809	0.926	-1.330 -0.901	0.686

q12 627.520 546	0.009	1.147	1.168	1.151	2.253	0.629

q13 432.947 546	1.000	0.791	0.844	-2.819 -2.645	0.753

q14 363.199 546	1.000	0.664	0.901	-2.101 -1.147	0.710

q15 436.098 546	1.000	0.797	0.875	-1.569 -1.714	0.726

q16 534.001 546	0.635	0.976	1.110	-0.130	1.270	0.612

q17 388.658 546	1.000	0.711	0.791	-1.939 -2.467	0.718

q18 397.202 546	1.000	0.726	0.794	-3.325 -3.077	0.745

q19 461.016 546	0.997	0.843	0.924	-1.928 -1.235	0.739

q20 731.609 546	0.000	1.337	1.324	2.763	4.549	0.583

q21 410.741 546	1.000	0.751	0.864	-2.731 -2.096	0.741

q22 524.738 546	0.736	0.959	1.033	-0.259	0.474	0.674

q23 736.580 546	0.000	1.347	1.158	1.407	1.166	0.451

q24 496.600 546	0.936	0.908	1.022	-0.402	0.272	0.642

q25 480.991 546	0.979	0.879	0.961	-0.892 -0.531	0.706

**Wald test on item level (z-values):**

z-statistic p-value

beta q1.c1	2.730	0.006

beta q1.c2	2.360	0.018

beta q1.c3	1.466	0.143

beta q1.c4	-0.599	0.549

beta q4.c1	3.213	0.001

beta q4.c2	1.061	0.289

beta q4.c3	-1.468	0.142

beta q4.c4	-1.186	0.236

beta q7.c1	1.365	0.172

beta q7.c2	2.376	0.018

beta q7.c3	1.383	0.167

beta q7.c4	0.582	0.561

beta q9.c1	1.860	0.063

beta q9.c2	1.640	0.101

beta q9.c3	-1.080	0.280

beta q9.c4	-1.182	0.237

beta q12.c1	-0.754	0.451

beta q12.c2	-1.075	0.282

beta q12.c3	-1.187	0.235

beta q12.c4	0.571	0.568

beta q13.c1	0.944	0.345

beta q13.c2	-2.128	0.033

beta q13.c3	-0.981	0.327

beta q13.c4	-1.084	0.278

beta q25.c1	-1.902	0.057

beta q25.c2	-1.978	0.048

beta q25.c3	-1.414	0.157

beta q25.c4	-0.546	0.585

Andersen LR-test

LR-value: 64.609

Chi-square df: 27

p-value: 0
