# Supplementary material for: Assessing maladaptive sugar-rich food consumption: development and preliminary validation of the sugar misuse questionnaire
Source: Front Nutr. 2026 Aug 7;13:1855019. doi: 10.3389/fnut.2026.1855019 (PMC13495693; doi:10.3389/fnut.2026.1855019)
Supplement: Supplementary file 1 [file Data_Sheet_1.zip › Appendix S1A.docx]

**Appendix S1A. Validated Sugar Misuse Questionnaire (SuMQ-Q11): Administration, Scoring and Interpretation Guide**

**This appendix describes the validated version of the Sugar Misuse Questionnaire (SuMQ-Q11), which should be used for administration, scoring and interpretation.**

**1. Purpose**

The Sugar Misuse Questionnaire (SuMQ) was developed to assess the severity of maladaptive patterns of sugar-rich food consumption in adults.

The questionnaire was developed using a sequential mixed-methods approach. Qualitative interviews first identified the principal experiential domains characterizing Sugar Misuse. These domains were subsequently translated into questionnaire items and psychometrically evaluated in a general population sample.

Psychometric analyses (Rasch modeling, principal component analysis and confirmatory factor analysis) demonstrated that an **11-item version (Q11)** provided the best psychometric performance. This validated version should therefore be used for routine administration, score calculation and interpretation.

The SuMQ is intended as a dimensional assessment instrument rather than a diagnostic tool.

**The questionnaire provides a single validated overall score reflecting the severity of Sugar Misuse.**

**2. Administration**

- Self-administered questionnaire
- Approximately 5 minutes
- Reference period: previous 12 months
- One response per item
- Response options scored from 0 (Never) to 4 (4 times a week or more)

**3. Scoring**

Each item is scored from **0 to 4**.

No reverse scoring is required.

The total SuMQ score is obtained by summing all 11 items.

Possible score range: **0–44**

Higher scores indicate greater severity of Sugar Misuse.

**All 11 items contribute equally to the total score. No weighting or transformation of item scores is required.**

**4. Interpretation**

| **Total score** | **Interpretation** |
| --- | --- |
| **0–13** | Absent to moderate Sugar Misuse |
| **14–17** | Preliminary severe Sugar Misuse |
| **18–44** | Preliminary very severe Sugar Misuse |

These thresholds were derived from ROC analyses using self-reported severity as the reference criterion.

They should be regarded as **preliminary research thresholds** rather than validated diagnostic cut-offs.

The SuMQ may currently be used to:

- quantify Sugar Misuse severity;
- compare participants in research studies;
- monitor changes over time;
- identify individuals who may benefit from further clinical assessment.

Future studies are required to establish validated diagnostic thresholds and normative values.

**Scores should always be interpreted in conjunction with clinical judgment and the participant’s overall clinical context.**

**5. Descriptive statistics from the validation sample**

To facilitate interpretation of the validated SuMQ score, descriptive statistics observed in the validation sample are presented below. These values are provided for descriptive purposes only and **should not be considered normative reference values or diagnostic thresholds**. They simply illustrate the distribution of validated SuMQ scores across the three self-reported severity groups used in the ROC analyses.

| **Validation sample subgroup** | **Mean ± SD** | **Median (Q1–Q3)** |
| --- | --- | --- |
| None to moderate Sugar Misuse | 4.82 ± 4.85 | 4 (2–7) |
| Severe Sugar Misuse | 20.04 ± 7.81 | 20 (14–25) |
| Very severe Sugar Misuse | 29.13 ± 8.86 | 29 (22–35) |

These descriptive statistics are intended to help users compare an individual’s score with the score distributions observed during the initial validation study. They are not intended to replace the validated Q11 scoring procedure or the preliminary thresholds presented above.

**6. Validated questionnaire (Q11)**

| **Item** | **The following questions refer to your behavior over the past 12 months.** | Never | Once a month or less | 2–4 times a month | 2–3 times a week | 4 times a week or more |
| --- | --- | --- | --- | --- | --- | --- |
| 1 | Did the consumption of sugar-rich products represent a moment of intense pleasure for you? | 0 | 1 | 2 | 3 | 4 |
| 3 | Did you use sugar-rich products to avoid episodes of anxiety or agitation? | 0 | 1 | 2 | 3 | 4 |
| 5 | Did you experience intense and/or urgent urges to consume sugar-rich products? | 0 | 1 | 2 | 3 | 4 |
| 8 | Did you consume sugar-rich products in larger amounts or over a longer period than you had intended? | 0 | 1 | 2 | 3 | 4 |
| 11 | Did you consume sugar-rich products in secret to avoid comments from people around you? | 0 | 1 | 2 | 3 | 4 |
| 13 | Did consuming sugar-rich products trigger the desire to consume even more? | 0 | 1 | 2 | 3 | 4 |
| 14 | In the absence of sugar-rich products, did you experience symptoms such as irritability, nervousness, agitation, anxiety, or physical discomfort? | 0 | 1 | 2 | 3 | 4 |
| 16 | Did people around you make comments about your consumption of sugar-rich products? | 0 | 1 | 2 | 3 | 4 |
| 18 | Did you experience uncontrollable urges to buy sugar-rich products? | 0 | 1 | 2 | 3 | 4 |
| 19 | Did the consumption of sugar-rich products lead to feelings of guilt? | 0 | 1 | 2 | 3 | 4 |
| 25 | Did you continue to consume sugar-rich products despite persistent or recurrent psychological or physical consequences that may have been caused or worsened by their consumption? | 0 | 1 | 2 | 3 | 4 |
