# Supplementary material for: Assessing maladaptive sugar-rich food consumption: development and preliminary validation of the sugar misuse questionnaire
Source: Front Nutr. 2026 Aug 7;13:1855019. doi: 10.3389/fnut.2026.1855019 (PMC13495693; doi:10.3389/fnut.2026.1855019)
Supplement: Supplementary file 1 [file Data_Sheet_1.zip › Appendix S1B.docx]

**Appendix S1B. Original Developmental Version of the Sugar Misuse Questionnaire (SuMQ-25)**

**Purpose**

The questionnaire presented below corresponds to the **original 25-item version** developed during the qualitative phase of instrument development.

This version is provided **for transparency and documentation of the instrument development process**.

The 25 items were generated from qualitative interviews and were originally organized around seven conceptual domains:

- Consequences
- Craving
- Loss of control
- Tolerance-like experiences
- Withdrawal-like experiences
- Predisposing factors
- Triggers

Subsequent psychometric analyses demonstrated that the validated scoring algorithm is based on the reduced **11-item version (Q11)**.

**Accordingly, the 25-item version should not be used for score calculation or interpretation.**

The 25-item version is provided solely to document the conceptual development of the questionnaire and to facilitate future methodological research.

**Researchers interested in the conceptual development of Sugar Misuse may nevertheless find this version useful for hypothesis generation or future psychometric studies.**

| **Item** | **The following questions refer to your behavior over the past 12 months.** | Never | Once a month or less | 2–4 times a month | 2–3 times a week | 4 times a week or more |
| --- | --- | --- | --- | --- | --- | --- |
| 1 | Did the consumption of sugar-rich products represent a moment of intense pleasure for you? | 0 | 1 | 2 | 3 | 4 |
| 2 | Did consuming sugar-rich products help improve your mood? | 0 | 1 | 2 | 3 | 4 |
| 3 | Did you use sugar-rich products to avoid episodes of anxiety or agitation? | 0 | 1 | 2 | 3 | 4 |
| 4 | Was the consumption of sugar-rich products an important source of satisfaction in your daily life? | 0 | 1 | 2 | 3 | 4 |
| 5 | Did you experience intense and/or urgent urges to consume sugar-rich products? | 0 | 1 | 2 | 3 | 4 |
| 6 | Did your consumption of sugar-rich products feel like a real need rather than a simple desire? | 0 | 1 | 2 | 3 | 4 |
| 7 | Did you consume sugar-rich products outside of meals? | 0 | 1 | 2 | 3 | 4 |
| 8 | Did you consume sugar-rich products in larger amounts or over a longer period than you had intended? | 0 | 1 | 2 | 3 | 4 |
| 9 | Did you continue to consume sugar-rich products even when you were no longer hungry? | 0 | 1 | 2 | 3 | 4 |
| 10 | Did you continue to consume sugar-rich products even when their taste was no longer appealing to you? | 0 | 1 | 2 | 3 | 4 |
| 11 | Did you consume sugar-rich products in secret to avoid comments from people around you? | 0 | 1 | 2 | 3 | 4 |
| 12 | Did you ever avoid having sugar-rich products available because you were afraid of not being able to control strong urges? | 0 | 1 | 2 | 3 | 4 |
| 13 | Did consuming sugar-rich products trigger the desire to consume even more? | 0 | 1 | 2 | 3 | 4 |
| 14 | In the absence of sugar-rich products, did you experience symptoms such as irritability, nervousness, agitation, anxiety, or physical discomfort? | 0 | 1 | 2 | 3 | 4 |
| 15 | Did you consume sugar-rich products to relieve or avoid symptoms such as irritability, nervousness, agitation, anxiety, or physical discomfort? | 0 | 1 | 2 | 3 | 4 |
| 16 | Did people around you make comments about your consumption of sugar-rich products? | 0 | 1 | 2 | 3 | 4 |
| 17 | Did you hide from the sight of others in order to consume sugar-rich products? | 0 | 1 | 2 | 3 | 4 |
| 18 | Did you experience uncontrollable urges to buy sugar-rich products? | 0 | 1 | 2 | 3 | 4 |
| 19 | Did the consumption of sugar-rich products lead to feelings of guilt? | 0 | 1 | 2 | 3 | 4 |
| 20 | Did your consumption of sugar-rich products occur at specific times of the day? | 0 | 1 | 2 | 3 | 4 |
| 21 | Did you consume sugar-rich products to cope with anxiety, stress, or a depressed mood? | 0 | 1 | 2 | 3 | 4 |
| 22 | Did you feel the need to keep sugar-rich products available or stocked? | 0 | 1 | 2 | 3 | 4 |
| 23 | Did you feel the need to go out at any time and/or travel a long distance to buy sugar-rich products because none were available? | 0 | 1 | 2 | 3 | 4 |
| 24 | Did the consumption of sugar-rich products cause you feelings of distress or suffering? | 0 | 1 | 2 | 3 | 4 |
| 25 | Did you continue to consume sugar-rich products despite persistent or recurrent psychological or physical consequences that may have been caused or worsened by their consumption? | 0 | 1 | 2 | 3 | 4 |
